# Exhausting circ_0136474 and Restoring miR-766-3p Attenuate Chondrocyte Oxidative Injury in IL-1β-Induced Osteoarthritis Progression Through Regulating DNMT3A

**DOI:** 10.3389/fgene.2021.648709

**Published:** 2021-05-21

**Authors:** Haiquan Zhu, Shaobo Zhu, Xiuchao Shang, Xiangsheng Meng, Sheng Jing, Li Yu, Yu Deng

**Affiliations:** ^1^Department of Emergency Surgery, The First People’s Hospital of Lianyungang, Lianyungang, China; ^2^Department of Emergency Surgery, The Affiliated Lianyungang Hospital of Xuzhou Medical University, Lianyungang, China; ^3^Department of Emergency Surgery, The Affiliated Hospital of Kangda College of Nanjing Medical University, Lianyungang, China; ^4^Department of Emergency Surgery, Lianyungang Clinical College of Nanjing Medical University, Lianyungang, China; ^5^Department of Orthopaedic Trauma and Microsurgery, Zhongnan Hospital of Wuhan University, Wuhan, China

**Keywords:** circ_0136474, miR-766-3p, DNMT3A, chondrocytes, osteoarthritis

## Abstract

Circular RNA circ_0136474 is a new contributor of human osteoarthritis (OA) by suppressing chondrocyte proliferation. However, its role and mechanism in OA chondrocyte injury remain ill defined. Herein, we performed real-time quantitative PCR to detect RNA expression of circ_0136474, microRNA (miR)-766-3p, and DNA methyltransferase 3A (DNMT3A) and utilized Western blotting to measure protein expression of DNMT3A, matrix metalloproteinase-1 (MMP1), MMP13, collagen II, proliferating cell nuclear antigen (PCNA) and B cell lymphoma (Bcl)-2, and Bcl-2-associated X protein (Bax). Direct interaction between miR-766-3p and circ_0136474 or DNMT3A was confirmed by bioinformatics algorithms, dual-luciferase reporter assay, and RNA immunoprecipitation. Functional experiments including cell counting kit-8 assay, flow cytometry, and special assay kits were employed to measure oxidative injury in interleukin (IL)-1β-induced OA-like chondrocytes. First, IL-1β administration induced cell viability inhibition, collagen II suppression, and promotion of MMP1 and MMP13 in human chondrocyte CHON-001 cells. Expression of circ_0136474 and DNMT3A was upregulated, and miR-766-3p was downregulated in human OA cartilages and IL-1β-induced CHON-001 cells. Functionally, both blocking circ_0136474 and upregulating miR-766-3p could rescue cell viability and levels of PCNA, Bcl-2, reduced glutathione (GSH), and total superoxide dismutase (SOD), and attenuate apoptosis rate and levels of Bax, reactive oxygen species (ROS), and lipid peroxidation malondialdehyde (MDA). Mechanically, circ_0136474 served as miR-766-3p sponge to govern miR-766-3p-targeted DNMT3A expression. Accidently, restoring DNMT3A counteracted the miR-766-3p upregulation role, and silencing miR-766-3p weakened circ_0136474 knockdown effect in IL-1β-induced CHON-001 cells. In conclusion, exhausting circ_0136474 could mitigate OA chondrocyte oxidative injury through regulating miR-766-3p/DNMT3A axis.

## Highlights

- circ_0136474 was upregulated and miR-766-3p was downregulated in OA cartilage specimens and IL-1β-induced OA model in chondrocytes.
- Blocking circ_0136474 and upregulating miR-766-3p rescued cell viability of IL-1β-induced human chondrocytes and attenuated apoptosis and oxidative stress.
- There was a direct interaction between circ_0136474 and miR-766-3p in regulating DNMT3A and IL-1β-induced chondrocytes.

## Introduction

Osteoarthritis (OA) is a multifactorial chronic disease in the joints and is characterized by the degradation of articular cartilage, debilitating pain, and loss of mobility (Nguyen et al., 2017). The most prevalent risk factor of OA incidence is age (Loeser et al., 2016). Epigenetics such as non-coding RNAs and DNA methylation have emerged as a new and important area of research on OA etiology and therapy by interplaying with genetics (Grandi and Bhutani, 2020; Rice et al., 2020). Circular RNAs (circRNAs) are a new class of non-coding RNAs characterized by covalently closed loop structures produced by back-splicing event (Kristensen et al., 2019). Different expression profiles and functional analysis of circRNAs have been carried out in human OA cartilages (Li H. et al., 2019; Wang et al., 2019). Upregulated and downregulated circRNAs participate in the occurrence and progression of OA by regulating multiple pathological processes, such as extracellular matrix (ECM) degradation, inflammation, and apoptosis (Yu and Sun, 2018).

Moreover, oxidative stress and either inflammation or aging stress response are highly interdependent and interconnected (Haigis and Yankner, 2010; Biswas, 2016), thus serving as emerging players in OA pathogenesis and treatment (Minguzzi et al., 2018; Ansari et al., 2020). CircRNAs have been on the way forward for being promising biomarkers with therapeutic potential in OA (Li et al., 2018). Furthermore, the competing endogenous RNA (ceRNA) network such as circRNA-microRNA (miRNA)–messenger RNA (mRNA) axis has been a possible mechanism and therapeutic target for OA (Kulcheski et al., 2016; Xiao et al., 2019). Circ_0136474 is a new contributor of human OA by suppressing chondrocyte proliferation (Li Z. et al., 2019); however, its role and the ceRNA pathway in OA chondrocytes injury remain largely uncovered.

Interleukin-1β (IL-1β) is one major inflammatory and catabolic cytokine in the pathophysiology of OA and could induce OA-like chondrocytes (Jenei-Lanzl et al., 2019). Moreover, circRNAs are abnormally expressed in IL-1β-induced mice OA chondrocytes (Zhou et al., 2018). MiRNA (miR)-766-3p is a newly identified suppressor of inflammatory response (Hayakawa et al., 2019), and it regulates chondrocyte integrity under IL-1β stimulation (Li et al., 2020). Thereby, we planned to investigate the role of circ_0136474 in oxidative damage of IL-1β-induced OA model in human chondrocytes, as well as to determine the circ_0136474/miR-766-3p interaction in regulating DNA methyltransferase 3A (DNMT3A). DNMT3A is involved in DNA methylation in the epigenetics (Jeffries, 2019; Miranda-Duarte et al., 2020).

## Materials and Methods

### Cartilage Tissues

Osteoarthritis cartilage tissues were obtained from 33 OA patients who underwent total knee replacement surgery at The First People’s Hospital of Lianyungang; another 33 normal cartilage tissues at knee joints were from trauma patients without previous history of OA or rheumatoid arthritis. Clinical characteristics of these subjects are listed in Table 1, and clinical samples were collected during January 2017 to December 2019. Informed consent was obtained from all tissue donors. The study was approved by the Ethics Committee of the First People’s Hospital of Lianyungang and performed according to the Declaration of Helsinki.

**TABLE 1:** Clinical characteristics of osteoarthritis (OA) patients and healthy controls.

Clinical characteristics	Patients (*n* = 33)	Healthy (*n* = 33)	*P*-value
Age, years	53 ± 10	48 ± 5	0.5441
**Sex**			
Male	19	18	0.8041
Female	14	15	
BMI, kg/m^2^	24.5 ± 2.7	23.4 ± 2.0	0.7657
**OA stage**			
Stage I	11	–	
Stage II	9	–	
Stage III	8	–	
Stage IV	5	–	

### Cell Culture and IL-1β Induction

Human cartilage cell line CHON-001 (#CRL-2846) was from American Type Culture Collection (ATCC; Manassas, VA, United States) and cultured in ATCC-formulated Dulbecco’s modified Eagle’s medium (ATCC) supplemented with 0.1 mg/ml of G418 (Genomeditech, Shanghai, China) and 10% heat-inactivated fetal bovine serum (R&D systems, Minneapolis, MN, United States). CHON-001 cells were kept in a 5% CO_2_ atmosphere at 37°C. When CHON-001 cells reached to a confluence of 70%, cell culture medium was changed with the medium containing 2, 5, 10, or 20 ng/ml of IL-1β for 24 h, and the medium containing 10 ng/ml of IL-1β for 6, 12, 24, and 48 h. The control group was CHON-001 cells without IL-1β treatment.

### RNA Isolation

Nuclear and cytoplasmic fractions of CHON-001 cells were separated with the NE-PER Nuclear and Cytoplasmic Extraction Reagents (Thermo Fisher Scientific, Waltham, MA, United States) following the manufacturer’s protocol. The RNA isolation from the nucleus, cytoplasm, and total cells, as well as tissues was performed using Norgen Total RNA Isolation Plus Micro Kits (Norgen Biotek, Thorold, Canada).

### Quantitative Real-Time PCR and Ribonuclease R Treatment

A part (2 μg) of the total RNA from CHON-001 cells was treated with 3 U of RNase R (Duma, Shanghai, China) for 30 min at 37°C, and another part of the total RNA untreated with RNase R served as the mock group. After that, RNA samples (500 ng) were subjected to reverse transcription using Superscript VILO cDNA master mix (Invitrogen, Carlsbad, CA, United States), and qPCR was carried out using the above *de novo* cDNA, Fast SYBR Green master mix (Applied Biosystems, Carlsbad, CA, United States) and special primer pairs for circ_0136474, miR-766-3p, DNMT3A, and ASH2-like histone lysine methyltransferase complex subunit (ASH2L). Besides, threshold cycle (Ct) of detected RNA was examined on ABI7500 qPCR instrument (Applied Biosystems) and standardized to the housekeeping gene glyceraldehyde-3-phosphate dehydrogenase (GAPDH) or U6 small nuclear RNA (U6) for quantification. The primer pairs are listed in Table 2.

**TABLE 2:** The sequences of oligonucleotides and primers.

Name	Sequence
si-circ_0136474	5′-AAUUCCCCCUGGUCAGGGUUCdTdT-3′
miR-766-3p mimic	5′-ACUCCAGCCCCACAGCCUCAGC-3′
anti-miR-766-3p	5′-GCUGAGGCUGUGGGGCUGGAGU-3′
si-NC	5′-UUCUCCGAACGUGUCACGUdTdT-3′
miR-NC mimic	5′-ACGUGACACGUUCGGAGAATT-3′
anti-miR-NC	5′-CAGUACUUUUGUGUAGUACAA-3′
circ_0136474 (133 nt)	Forward primer 5′ACAGAAGTGGATGGGAGGC-3′ Reverse primer 5′-CCTTCTTGGTGGTCCCTGT-3′
ASH2-like histone lysine methyltransferase complex subunit (ASH2L) (140 nt)	Forward primer 5′ATGCAACAGGGGCAGAAGAG-3′ Reverse primer 5′ATCGACCAAGTTTGCCTCCC-3′
miR-766-3p (75 nt)	Forward primer 5′-ACTCCAGCCCCACAGCC-3′ Reverse primer 5′-GAACATGTCTGCGTATCTC-3′
DNA methyltransferase 3A (DNMT3A) transcript variant 2 (72 nt)	Forward primer 5′-GGTTGTGAGAAGGAATGGGCG-3′ Reverse primer 5′TTGGCTTTCTTCTCAGCCGTAT-3′
Glyceraldehyde-3-phosphate dehydrogenase (GAPDH) (104 nt)	Forward primer 5′-GACAGTCAGCCGCATCTTCT-3′ Reverse primer 5′GCGCCCAATACGACCAAATC-3′
U6 (80 nt)	Forward primer 5′CTCGCTTCGGCAGCACA-3′ Reverse primer 5′AACGCTTCACGAATTTGCGT-3′

### Cell Counting Kit-8 Assay

CHON-001 cells and IL-1β-treated CHON-001 cells were re-inoculated in a 96-well plate at a density of 3,000 cells per well. These cells were cultured in complete medium for another 0, 24, 48, and 72 h, and 10% (v/v) CCK-8 reagent (Genomeditech) was added in each well for another 2 h. Later, optical density (OD) values at 450 nm were measured on a microplate reader (Bio-Rad, Hercules, CA, United States). Cell viability of IL-1β-treated CHON-001 cells was normalized to the control cells (without IL-1β treatment) by calculating 100% × OD_*IL–*1β group_/OD_*Control group*_.

### Protein Extraction and Western Blotting

Total protein from tissues and CHON-001 cells was extracted from tissues and cells by radioimmunoprecipitation assay (RIPA; Beyotime, Shanghai, China) containing phenylmethylsulfonyl fluoride (PMSF; Beyotime). Next, protein samples were subjected to standard Western blotting as previously described (Li Z. et al., 2019). Briefly, protein separation was performed by sodium dodecyl sulfate-polyacrylamide gel electrophoresis, and protein transferring was conducted onto polyvinylidene difluoride membrane (Millipore, Bedford, MA, United States). Membranes were blocked by 5% non-fat milk, incubated with special primary antibodies and horseradish peroxidase-conjugated secondary antibodies, and last developed by enhanced chemiluminescence kit (Millipore). The antibodies are summarized in Table 3. The relative protein expression was normalized to β-actin and presented as fold change of that in the control group.

**TABLE 3:** The antibodies used in Western blotting.

Name	Cat. No.	Source
DNMT3A	20954-1-AP	Proteintech, Wuhan, China
MPP1	26585-1-AP	Proteintech
MPP13	18165-1-AP	Proteintech
Collagen II	AF6528	Beyotime, Shanghai, China
β-actin	AF0003	Beyotime
PCNA	10205-2-AP	Proteintech
Bcl-2	AB112	Beyotime
Bax	AF0057	Beyotime
HRP-rabbit IgG	SA00001-2	Proteintech
HRP-mouse IgG	SA00001-1	Proteintech

### Cell Transfection

Small interfering RNA targeting circ_0136474 (si-circ_0136474) and pcDNA4.1 vector (BioVector, Beijing, China) recombined with DNMT3A cDNA (pcDNA-DNMT3A) were used to silence circ_0136474 and overexpress DNMT3A, respectively. For miR-766-3p overexpression and knockdown, the mimic and inhibitor were synthesized. Above dysregulations in CHON-001 cells were performed using transient transfection using Lipofectamine 2000 (Invitrogen, Carlsbad, CA, United States) following the manufacturer’s protocol. The negative controls were si-NC, miR-NC mimic and inhibitor, as well as the empty pcDNA plasmid. These oligonucleotides are listed in Table 2. After transfection for 48 h, CHON-001 cells were treated with 0 and 10 ng/mL of IL-1β for 24 h prior to functional analysis.

### Flow Cytometry

Apoptosis of CHON-001 cells after IL-1β treatment was detected by the fluorescein isothiocyanate (FITC) Annexin V Apoptosis Detection Kit (BD Biosciences, San Jose, CA, United States). Cells were reaped with the scraper and washed with cold phosphate-buffer saline; then 1 × 10^5^ cells were re-suspended in 1 × Annexin V Binding Buffer and added with 5 μl of FITC annexin V and 5 μl of propidium iodide (PI) staining solution for 15 min at room temperature free from light. Eventually, these staining cells were diluted in 400 μl of 1 × annexin V binding buffer and analyzed on a flow cytometer (BD Biosciences). The percentage of early apoptotic cells and late apoptotic cells was considered as apoptosis rate (%).

### Reactive Oxygen Species, Reduced Glutathione, Malondialdehyde, and Superoxide Dismutase Assay Kits

Reactive oxygen species in CHON-001 cells was detected by 2′,7′-dichlorofluorescein diacetate (DCFH-DA) probe (Beyotime), and briefly, 5 × 10^6^ cells were incubated in serum-free medium containing 10 μM of DCFH-DA for 20 min. The fluorescence was determined on a microplate reader (Bio-Rad) with 488-nm excitation wavelength and 525-nm emission wavelength. In addition, GSH, MDA, and SOD levels in cell extracts were severally tested by GSH and Oxidized Glutathione Disulfide (GSSG) Assay Kit (Beyotime), Lipid Peroxidation MDA Assay Kit (Beyotime), and Total SOD Assay Kit with WST-8 (Beyotime) according to the manufacturer’s instructions.

### Dual-Luciferase Reporter Assay and RNA Immunoprecipitation

The wild type circ_0136474 (WT-circ_0136474) and its mutant derivative lacking the miR-766-3p binding site (MUT-circ_0136474) were synthesized and inserted into pGL3 luciferase report vectors (containing *Firefly*; Promega, Madison, WI, United States) downstream of the luciferase-coding region. Similarly, pGL3-WT-DNMT3A 3′UTR vector and pGL3-MUT-DNMT3A 3′UTR vector were constructed. Then CHON-001 cells were co-transfected with the abovementioned recombinant vectors and miR-766-3p mimic or miR-NC mimic, as well as pRL-TK luciferase report vector (containing *Renilla*; Promega). A dual-luciferase reporter assay system (Promega) was applied to analyze the luciferase activities at 48-h post-transfection with normalization to *Renilla*. RIP assay was performed using EZ-Magna RIP RNA-Binding Protein Immunoprecipitation Kit (Millipore); during this experiment, anti-argonaute 2 (anti-AGO2) or anti-IgG (negative control) were used to label magnetic beads, which were then incubated with cell extract of CHON-001 cells overnight at 4°C. The RNA–protein complex bound to the beads was lysed in Norgen Total RNA Isolation Plus Micro Kits (Norgen Biotek) and qPCR.

### Statistical Analysis

All experiments were repeated at least three times, and measured data were expressed as mean ± standard error of the mean in one representative experiment. Data were analyzed, and comparisons were measured on GraphPad Prism 7 (GraphPad, La Jolla, CA, United States). *P* < 0.05 was considered to be of statistical significance.

## Results

### Circ_0136474 Was an Upregulated circRNA in Osteoarthritis Patients

**FIGURE 1:**
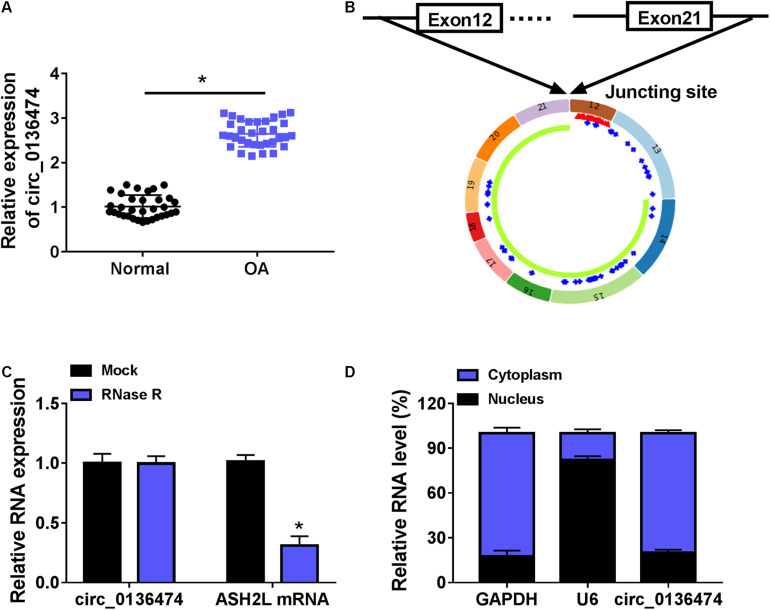
Circ_0136474 was an upregulated circRNA in osteoarthritis (OA) patients. **(A)** Quantitative real-time (qPCR) detected relative expression of circ_0136474 in OA cartilages (*n* = 33) and normal cartilages (*n* = 33). **(B)** Schematic diagram showing the junction site of exons 12–21 of ASH2L. **(C,D)** qPCR compared the relative RNA expression levels of circ_0136474, ASH2-like histone lysine methyltransferase complex subunit (ASH2L), glyceraldehyde-3-phosphate dehydrogenase (GAPDH), and U6 in human CHON-001 chondrocytes. *P < 0.05 was determined by two-way ANOVA or unpaired *t*-test.

Compared with normal cartilages, circ_0136474 was highly expressed in human OA cartilages with a back-splicing event of a junction site from exons 12–21 of ASH2L (Figures 1A,B). Moreover, its expression was little affected by RNase R treatment, whereas the host gene ASH2L mRNA expression was dramatically reduced by RNase R (Figure 1C). Circ_0136474 level was dominantly discovered in the cytoplasm of CHON-001 cells, which was paralleled with GAPDH and contrary to U6 (Figure 1D). These data showed that circ_0136474 was an abnormally upregulated circRNA in OA.

### IL-1β Induced Cell Viability Inhibition and Extracellular Matrix Degradation in CHON-001 Cells

**FIGURE 2:**
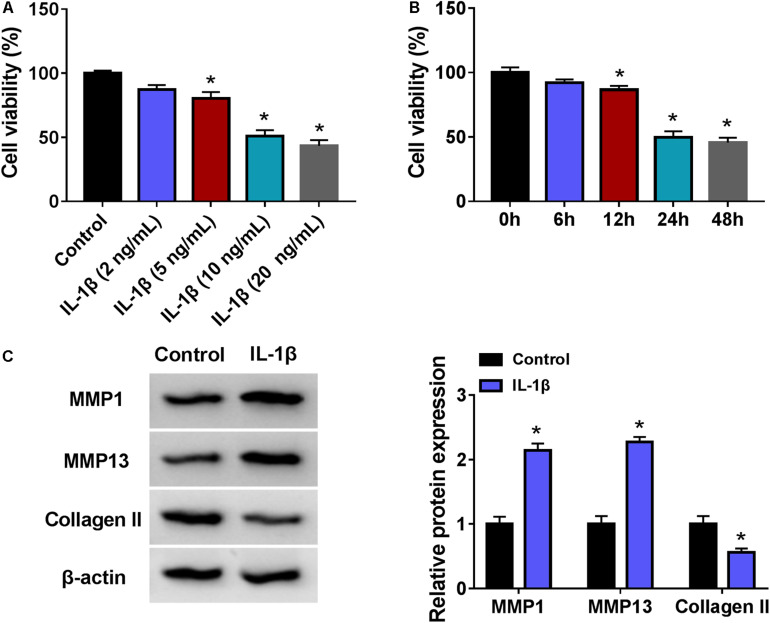
Interleukin-1β (IL-1β) induced OA model in human chondrocytes. **(A,B)** Cell counting kit (CCK-8) assay measured cell viability (%) of CHON-001 cells treated with **(A)** different concentrations of IL-1β and **(B)** 10 ng/ml of IL-1β for different times. **(C)** Western blotting examined relative protein expression of matrix metalloproteinase-1 (MMP1), MMP13, and Collagen II in CHON-001 cells with and without 10 ng/ml of IL-1β treatment for 24 h. *P < 0.05 was determined by ordinary one-way ANOVA or two-way ANOVA.

Interleukin-1β administration induced cell viability inhibition in CHON-001 cells in a certain of concentration- and time-dependent manner (Figures 2A,B); 10 ng/ml of IL-1β caused about 50% cell viability inhibition at 24 h (Figure 2B), and accompanied with high expression of matrix metalloproteinase-1 (MMP1) and MMP13 and low expression of collagen II (Figure 2C). These data demonstrated that IL-1β induced cell viability inhibition and ECM degradation in CHON-001 cells. OA was a disease of the cartilage pericellular matrix (Guilak et al., 2018), and thus, these data suggested a success of OA cell model in chondrocytes.

### Exhausting circ_0136474 Mitigated Interleukin-1β-Induced Apoptosis and Oxidative Stress in Chondrocytes

**FIGURE 3:**
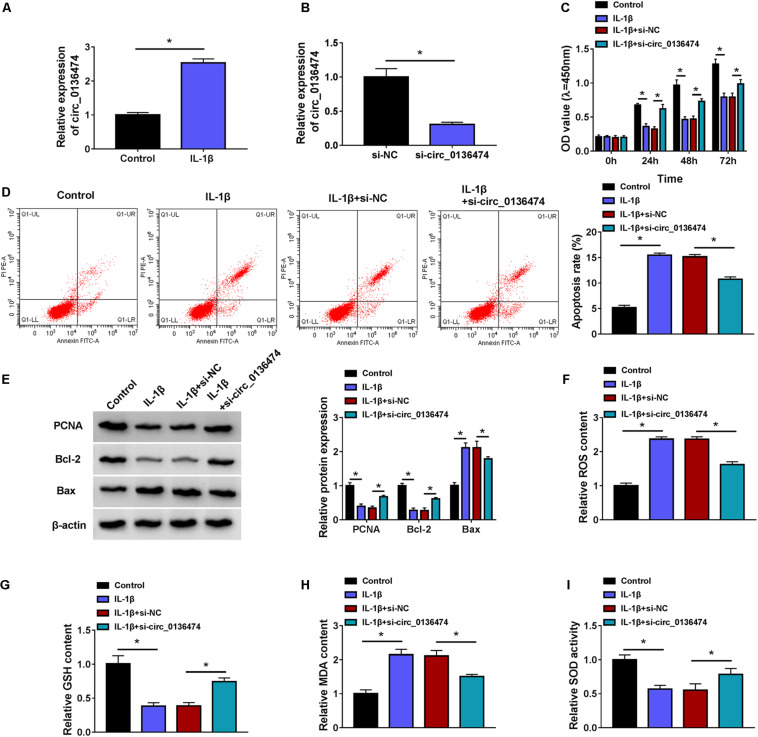
Exhausting circ_0136474 mitigated apoptosis and oxidative stress in IL-1β-induced chondrocytes. **(A)** qPCR detected relative expression of circ_0136474 in CHON-001 cells treated with 10 ng/ml of IL-1β for 24 h. IL-1β-induced CHON-001 cells were pre-transfected with si-circ_0136474/NC, **(B)** qPCR detected the relative expression of circ_0136474, **(C)** CCK-8 monitored the optical density (OD) values of inoculated cells during 72 h, **(D)** Flow cytometry (FCM) tested the apoptosis rate (%), **(E)** Western blotting examined the relative protein expression of proliferating cell nuclear antigen (PCNA), B cell lymphoma (Bcl)-2, and Bcl-2-associated X protein (Bax), and **(F–I)** special assay kits determined the levels of reactive oxygen species (ROS), reduced glutathione (GSH), malondialdehyde (MDA), and superoxide dismutase (SOD). *P < 0.05 was determined by unpaired *t*-test, ordinary one-way ANOVA, or two-way ANOVA.

Expression of circ_0136474 was increased in 10 ng/ml of IL-1β-induced CHON-001 cells (Figure 3A), and si-circ_0136474 pre-transfection could decrease this high level of circ_0136474 (Figure 3B). The inhibition of cell viability in IL-1β-disposed CHON-001 cells was saved with circ_0136474 exhaustion via transfection, as evidenced by the higher OD values during 72 h (Figure 3C). Apoptosis rate of CHON-001 cells was highly induced by IL-1β stimulation, and it could be partially attenuated by pre-transfecting si-circ_0136474 (Figure 3D). Molecularly, accompanied with cell viability promotion and apoptosis inhibition, elevated PCNA and Bcl-2 levels and attenuated Bax level were discovered in circ_0136474-silenced CHON-001 cells under IL-1β stress (Figure 3E). Besides, oxidative stress-related factors ROS and MDA were upregulated, while GSH and SOD were downregulated in CHON-001 cells in response to IL-1β treatment (Figures 3F–I); surprisingly, silencing circ_0136464 could overall counteract these oxidative stress levels (Figures 3F–I). These results revealed that exhausting circ_0136474 was responsible to attenuate apoptosis and oxidative stress in IL-1β-induced OA model in human chondrocytes.

### Circ_0136474 Could Target miR-766-3p in Human Chondrocytes

**FIGURE 4:**
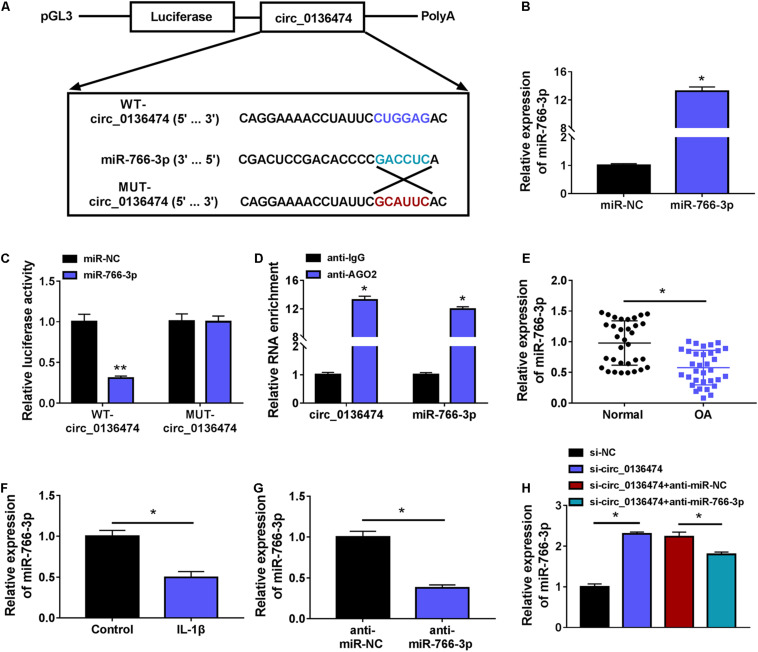
Circ_0136474 could target miR-766-3p in human chondrocytes. **(A)** Schematic diagram showed the construction of pGL3 luciferase report vectors carrying WT-circ_0136474 or MUT-circ_0136474. **(B)** qPCR detected the relative expression of miR-766-3p in CHON-001 cells transfected with miR-766-3p/NC mimic (miR-766-3p/NC). **(C)** The dual-luciferase reporter assay measured the relative luciferase activity of WT-circ_0136474 and MUT-circ_0136474 vectors with co-transfection of miR-766-3p/NC. **(D)** The radioimmunoprecipitation assay (RIP) assay confirmed the relative RNA enrichments of circ_0136474 and miR-766-3p in CHON-001 cells. **(E–H)** qPCR detected the relative miR-766-3p expression in **(E)** OA cartilages (*n* = 33) and normal cartilages (*n* = 33), **(F)** control CHON-001 cells and IL-1β (10 ng/m-)-induced CHON-001 cells, **(G,H)** IL-1β (10 ng/ml)-induced CHON-001 cells pre-transfected with anti-miR-766-3p/NC, si-circ_0136474/NC, or si-circ_0136474 combined with anti-miR-766-3p/NC. *P < 0.05 and **P < 0.01 were determined by unpaired *t-*test, ordinary one-way ANOVA, or two-way ANOVA.

According to the prediction of circinteractome, we hypothesized that there might be a novel target binding interaction between circ_0136474 and miR-766-3p. To further validate this hypothesis, pGL3-WT-circ_0136474 vector and pGL3-MUT-circ_0136474 vector were constructed (Figure 4A). As a result, miR-766-3p overexpression via mimic transfection resulted in a loss of luciferase activity of pGL3-WT-circ_0136474 vector in CHON-001 cells, whereas there was no deficit of that with the pGL3-MUT-circ_0136474 vector (Figures 4B,C). Furthermore, circ_0136474 and miR-766-3p were co-enriched in AGO2 RIP with normalization to IgG RIP (Figure 4D). In OA, miR-766-3p expression was lower in human OA cartilages and CHON-001 chondrocytes under IL-1β (10 ng/ml) stress (Figures 4E,F). Collectively, miR-766-3p could be targeted by circ_0136474 and downregulated in human OA.

### Blocking miR-766-3p Blocked the Suppressive Role of circ_0136474 Knockdown in Interleukin-1β-Induced Oxidative Injury in Chondrocytes

**FIGURE 5:**
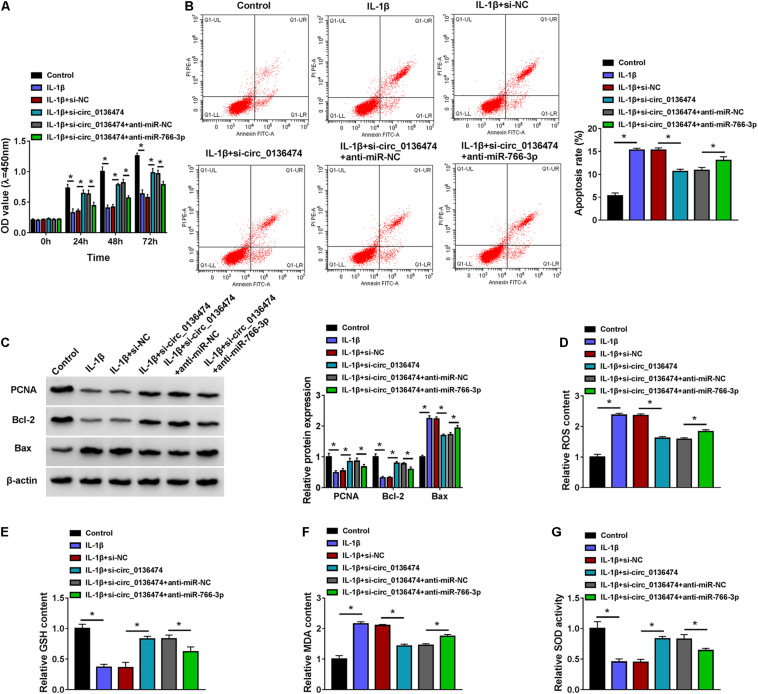
Blocking miR-766-3p could block the role of circ_0136474 knockdown in IL-1β-induced chondrocytes. CHON-001 cells were transfected with si-circ_0136474/NC or si-circ_0136474 combined with anti-miR-766-3p/NC prior to treatment of IL-1β (10 ng/ml) for 24 h. **(A)** CCK-8 monitored the OD values of inoculated cells during 72 h. **(B)** FCM tested the apoptosis rate (%). **(C)** Western blotting examined the relative protein expression of PCNA, Bcl-2, and Bax. **(D–G)** Special assay kits determined the levels of ROS, GSH, MDA, and SOD. *P < 0.05 was determined by ordinary one-way ANOVA or two-way ANOVA.

Additionally, circ_0136474 knockdown in 10 ng/ml of IL-1β-induced CHON-001 cells mediated miR-766-3p upregulation (Figure 4H), and this upregulation was then weakened by blocking miR-766-3p via miR-766-3p inhibitor (anti-miR-766-3p) co-transfection (Figures 4G,H). What is more, cell viability and apoptosis rate of IL-1β-induced CHON-001 cells were, respectively, promoted and suppressed by silencing circ_0136474, and these effects were both attenuated by silencing miR-766-3p along with circ_0136474 (Figures 5A,B). Circ_0136474 depletion led to upregulation of proliferation/apoptosis-related proteins PCNA and Bcl-2 accompanying Bax downregulation, which was overall blocked by depleting miR-766-3p (Figure 5C). Contents of ROS and MDA in circ_0136474-silenced CHON-001 cells under IL-1β stress were facilitated with addition of anti-miR-766-3p, and GSH and SOD were diminished (Figures 5D–G). These outcomes demonstrated that blocking miR-766-3p could block the suppressive role of circ_0136474 knockdown in IL-1β-induced oxidative injury in chondrocytes, suggesting a circ_0136474/miR-766-3p interaction in regulating OA-like chondrocyte oxidative injury.

### DNMT3A Was a Target Gene for circ_0136474/miR-766-3p

**FIGURE 6:**
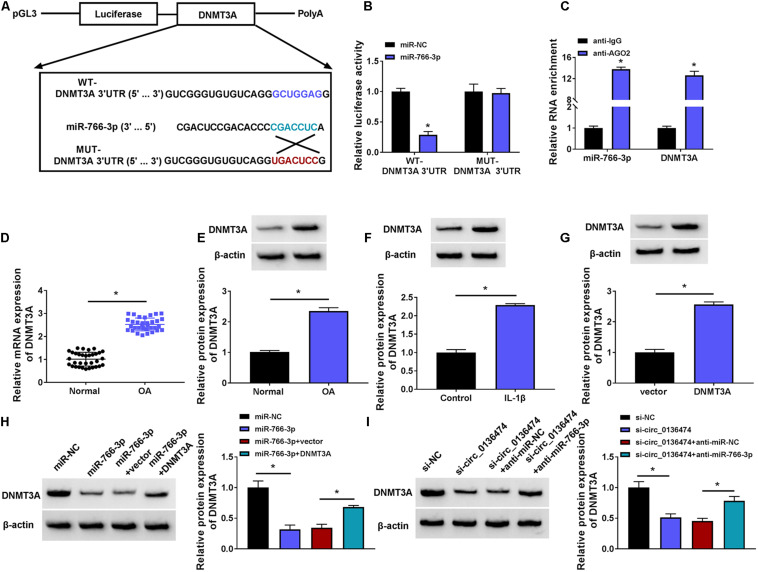
DNMT3A was a target gene for circ_0136474/miR-766-3p. **(A)** Schematic diagram showing the construction of pGL3 luciferase report vectors carrying WT-DNMT3A 3’UTR or MUT-DNMT3A 3’UTR. **(B)** Dual-luciferase reporter assay measured the relative luciferase activity of WT-DNMT3A 3’UTR and MUT-DNMT3A 3’UTR vectors with co-transfection with miR-766-3p/NC in CHON-001 cells. **(C)** RIP assay confirmed the relative RNA enrichments of DNMT3A and miR-766-3p in CHON-001 cells. **(D)** qPCR detected the relative mRNA expression of DNMT3A, and **(E–I)** Western blotting examined the relative protein expression of DNMT3A in **(D,E)** OA cartilages (*n* = 33) and normal cartilages (*n* = 33), **(F)** control CHON-001 cells and IL-1β (10 ng/ml)-induced CHON-001 cells, **(G–I)** IL-1β (10 ng/ml)-induced CHON-001 cells pretransfected with DNMT3A vector or empty pcDNA vector (vector), miR-766-3p/NC or miR-766-3p combined with DNMT3A or vector, si-circ_0136474/NC or si-circ_0136474 combined with anti-miR-677-3p or anti-miR-NC. *P < 0.05 was determined by unpaired *t*-test, ordinary one-way ANOVA or two-way ANOVA.

Similarly, DNMT3A was predicted to contain miR-766-3p binding sites according to the TargetScan database. Here, DNMT3A was selected as a candidate target for miR-766-3p to be further clarified (Figure 6A). The luciferase activity of pGL3-WT-DNMT3A 3′UTR vector was impaired by miR-766-3p mimic, and pGL3-MUT-DNMT3A 3′UTR vector was unacted by miR-766-3p (Figure 6B). RIP assay indicated a co-enrichment of miR-766-3p and DNMT3A in anti-AGO2-mediated precipitated complex (Figure 6C). Expression of DNMT3A was increased in OA cartilage specimens and IL-1β (10 ng/ml)-induced OA model in CHON-001 cells (Figures 6D–F). In response to miR-766-3p mimic transfection, DNMT3A protein expression was extremely depressed in IL-1β-induced CHON-001 cells (Figure 6H); nevertheless, this DNMT3A depression could be salvaged by co-transfection of pcDNA-DNMT3A (DNMT3A) vector (Figures 6G,H). Besides, si-circ_0136474 transfection either led to low DNMT3A in IL-1β-induced CHON-001 cells, and this was rescued by co-transfecting anti-miR-766-3p as well (Figure 6I). Thus, DNMT3A could be a target gene for circ_0136474/miR-766-3p in human OA chondrocytes.

### Restoring miR-766-3p Attenuated Interleukin-1β-Induced Oxidative Injury in Chondrocytes and This Effect Was Abrogated by DNA Methyltransferase 3A Upregulation

**FIGURE 7:**
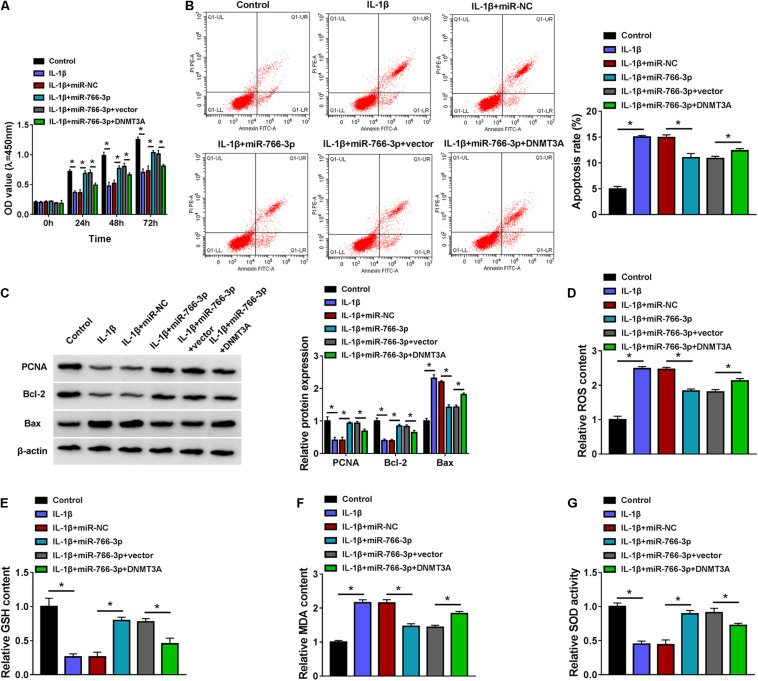
Restoring miR-766-3p attenuated IL-1β-induced oxidative injury in chondrocytes, and this effect was abrogated by DNMT3A upregulation. CHON-001 cells were transfected with miR-766-3p/NC or miR-766-3p combined with DNMT3A or vector prior to treatment of IL-1β (10 ng/ml) for 24 h. **(A)** CCK-8 monitored the OD values of inoculated cells during 72 h. **(B)** FCM tested the apoptosis rate (%). **(C)** Western blotting examined the relative protein expression of PCNA, Bcl-2, and Bax. **(D–G)** Special assay kits determined the levels of ROS, GSH, MDA, and SOD. *P < 0.05 was determined by ordinary one-way ANOVA or two-way ANOVA.

Re-expression of miR-766-3p via mimic transfection could improve cell viability of IL-1β-induced CHON-001 cells and suppress apoptosis rate (Figures 7A,B). IL-1β-stimulated high expression levels of Bax, ROS, and MDA (10 ng/ml) were attenuated by introducing miR-766-3p mimic (Figures 7C,D,F), along with ameliorative levels of PCNA, Bcl-2, GSH, and SOD (Figures 7C,E,G). Notably, these effects of miR-766-3p upregulation were lowered in the co-presence of DNMT3A vector (Figures 7A–G). Therefore, restoring miR-766-3p also suppressed IL-1β-induced oxidative injury in human chondrocytes, and this protective effect could be abrogated by DNMT3A upregulation, implying an miR-766-3p/DNMT3A axis in regulating IL-1β-induced OA chondrocyte oxidative injury.

## Discussion

Oxidative stress and ROS overproduction played an important role in the pathogenesis of OA, and IL-1β could induce oxidative damage in both articular chondrocytes and synovial fibroblasts (Zhang et al., 2018; Bao et al., 2020). Furthermore, oxidative stress, epigenetic changes, including miRNA regulation, were suggested as potential therapeutic targets in OA (Portal-Nunez et al., 2016). However, the role of circRNAs and long non-coding RNAs that could function as an miRNA sponge was still unknown in oxidative stress injury in osteoarthritic chondrocytes. Thus, we focused on investigating circ_0136474 dysregulation, role, and ceRNA mechanism in IL-1β-induced apoptosis and oxidative stress.

In this study, we observed an upregulation of circ_0136474 in human OA cartilage tissues and chondrocytes under IL-1β stimulation, and this finding was consistent with that in primary OA chondrocytes isolated from patients (Li Z. et al., 2019). IL-1β could induce an OA cell model *in vitro*, as evidenced by cell viability inhibition and ECM degradation in IL-1β-exposed CHON-001 cells. Functionally, blocking circ_0136474 could neutralize oxidative stress and apoptosis in IL-1β-induced chondrocytes by scavenging ROS and MDA, promoting cell proliferation and activating GSH and SOD. Among these chondroprotective effects of circ_0136474 knockdown, cell proliferation promotion and apoptosis suppression had been declared by Li Z. et al. (2019); however, the anti-oxidative stress role of exhausting circ_0136474 might be a new finding in OA pathogenesis and treatment. Besides, expression of circ_0136474 was predominantly detected in the cytoplasm instead of the nucleus in human chondrocytes, suggesting a latent potential of circ_0136474 as an endogenous miRNA sponge. Furthermore, we tested this hypothesis and validated that miR-766-3p was a novel target for circ_0136474, except for the previously reported miR-127-5p (Li Z. et al., 2019).

According to this research, miR-766-3p was abnormally downregulated in human OA cartilages and IL-1β-induced OA model in chondrocytes. On one hand, lower miR-766-3p could abate the chondroprotective effect of circ_0136474 deficiency in IL-1β-induced chondrocytes; on the other hand, high miR-766-3p attenuated IL-1β-induced cell proliferation inhibition, apoptosis, and oxidative stress, implying that miR-766-3p overexpression could mimic the chondroprotective effect of circ_0136474 deficiency. Furthermore, inhibiting circ_0136474 and re-expressing miR-766-3p might also be therapeutic approaches to antagonize OA progression. Additionally, the suppressive role of miR-766-3p in IL-1β-induced apoptosis and ECM degradation had been previously demonstrated (Li et al., 2020). Besides, the antioxidant property of miR-766-3p, it was also reported that a higher ROS was discovered to be paralleled with downregulation of a panel of five miRNAs including miR-766-3p in patients with low intrafollicular fluid melatonin (Khan et al., 2020). Even though miR-766-3p was considered as an anti-inflammatory miRNA in IL-1β-blamed and tumor necrosis factor-α (TNF-α)-blamed synoviocyte fibroblasts (Hayakawa et al., 2019), inflammatory response had not been further confirmed in this study.

We identified DNMT3A as a novel functional target gene for miR-766-3p. In addition, DNMT3B was confirmed to be directly regulated by miR-766-3p several years ago (Afgar et al., 2016), and DNA methylation of some tumor suppressors such as DKK2 could be decreased by transfecting miR-766-3p-expressing viruses. Notably, miR-766-3p was obviously downregulated in human tumors (You et al., 2018; Zhang et al., 2020), and its expression could be regulated by DNA methylation (Chen et al., 2017). DNMT3A expression was elevated in OA cartilages and OA-like chondrocytes, which supported the previous data (Wu et al., 2017; Ma et al., 2019). This high DNMT3A could functionally alleviate miR-766-3p overexpression-mediated chondroprotective effect under IL-1β stimulation by promoting apoptosis and oxidative stress levels. Notably, Vrtacnik et al. (2018) presently indicated that gene expression of epigenetic enzymes including DNMT3A was affected by oxidative stress and hypoxia *in vitro* and in patients with postmenopausal osteoporosis and OA. Moreover, the correlation between DNMT3A and oxidative stress had also been wildly spread in diverse cells (Maugeri et al., 2018; El Henafy et al., 2020; Jung et al., 2020). Apart from modulating proliferation, apoptosis, and oxidative stress, DNMT3A was previously demonstrated to be implicated in ECM destruction in OA (Wu et al., 2017). In addition, Wu et al. (Chen et al., 2017) claimed that the promoter of miR-766-3p was highly methylated in renal cell carcinoma tissues, suggesting that DNA methylation could, in turn, control miR-766-3p expression as well. Plus, PPARγ played a key role in OA development, and its promoter hypermethylation was attributed to DNMT3A and DNMT1 (Zhu et al., 2019). These finding suggested a possible regulatory back loop of miR-766-3p/DNMT3A in OA chondrocytes; however, this hypothesis was left to be further deciphered, as well as the discovery of DNMT3A downstream targets.

## Conclusion

We demonstrated the chondroprotective effects of circ_0136474 exhaustion and miR-766-3p upregulation against IL-1β-induced oxidative stress by suppressing DNMT3A. This study suggested a possible circ_0136474/miR-766-3p/DNMT3A ceRNA axis in IL-1β-induced OA pathogenesis and treatment.

## Data Availability Statement

The datasets used and/or analyzed during the current study are available from the corresponding author on reasonable request.

## Ethics Statement

The studies involving human participants were reviewed and approved by The First People’s Hospital of Lianyungang. The patients/participants provided their written informed consent to participate in this study.

## Author Contributions

HZ designed the study and wrote the manuscript. SZ and XS analyzed the data. XM and SJ performed the experiments. LY and YD summarized the data. All authors contributed to this study, and read and approved the manuscript.

## Conflict of Interest

The authors declare that the research was conducted in the absence of any commercial or financial relationships that could be construed as a potential conflict of interest.
